# Snazer: the simulations and networks analyzer

**DOI:** 10.1186/1752-0509-4-1

**Published:** 2010-01-07

**Authors:** Tommaso Mazza, Gennaro Iaccarino, Corrado Priami

**Affiliations:** 1 The Microsoft Research University of Trento, CoSBi, Trento, Italy; 2 DISI - University of Trento, Trento, Italy

## Abstract

**Background:**

Networks are widely recognized as key determinants of structure and function in systems that span the biological, physical, and social sciences. They are static pictures of the interactions among the components of complex systems. Often, much effort is required to identify networks as part of particular patterns as well as to visualize and interpret them.

From a pure dynamical perspective, simulation represents a relevant *way*-*out*. Many simulator tools capitalized on the "noisy" behavior of some systems and used formal models to represent cellular activities as temporal trajectories. Statistical methods have been applied to a fairly large number of replicated trajectories in order to infer knowledge.

A tool which both graphically manipulates reactive models and deals with sets of simulation time-course data by aggregation, interpretation and statistical analysis is missing and could add value to simulators.

**Results:**

We designed and implemented *Snazer*, the simulations and networks analyzer. Its goal is to aid the processes of visualizing and manipulating reactive models, as well as to share and interpret time-course data produced by stochastic simulators or by any other means.

**Conclusions:**

*Snazer *is a solid prototype that integrates biological network and simulation time-course data analysis techniques.

## Background

Proteins and genes play fundamental roles in most organic functions. They cooperate to keep the entire organism's machinery working and prevent breakdowns. Globally, their social relationships give rise to minutely organized networks, currently the target of meticulous studies [1]. Genetic Regulatory Networks (GRNs) [2,3] and Protein-to-Protein Interaction networks (PPIs) are the most representative classes of biological networks.

GRNs represent collections of DNA segments which functionally interact with each other as well as with the chemicals that govern the transcription of genes into RNA sequences. From another perspective, GRNs can be seen as input-output machineries which produce an output (i.e. the expression level of a gene) by a combined application of basic functions to input stimuli. PPIs are comprised of proteins that form chains in which each protein reacts to a stimulus of its predecessor to produce a signal directed to its successor. Reactions in such chains are usually seen as directionally oriented because often they are not reversible. A reaction chain acts as a connector of a triggering event to a final physiological response and then sets up a complex (and often cyclic) collaborative network.

Both kinds of networks have been collected into several data banks spread throughout the WWW [4] and modeled in a computerized fashion by means of some unambiguous and artificial formalisms. The most common one makes use of coupled Ordinary Differential Equations (ODEs) [5]. Alternatively, several other promising modeling techniques have been employed: (including) Boolean networks [6], Petri nets [7], Bayesian networks [8], graphical Gaussian models [9], Process Calculi [10,11] and Automata Theory [12]. Standard languages, xml- (SBML [13], CellML [14]) or graphical- (SBGN [15], BlenX4Bio [16]) based, have been further proposed to allow knowledge sharing. All of them are (roughly) connected to graph theory, since a common simple principle holds: interacting agents (e.g. genes, proteins, enzymes, etc.) are represented as the graph vertices whereas interactions (e.g dimerization, phosphorylation, collision, etc.) constitute the graph edges. Moreover, strength of connections (if any) is usually modeled by weighing the edges (e.g. to quantitatively represent fluxes in metabolic networks). Protein-to-protein interaction networks, chemical structures, gene co-expression and contact graphs for protein structures are all examples of undirected graphs, whereas experimental protocols and taxonomies of species and traditional regulatory networks are examples of directed graphs. Other examples include: DNA, RNA or protein sequences (linear graphs), sequence fragment overlap graphs (interval graphs) for shotgun sequence assembly, genetic maps and multiple sequence alignments (partial orders). On top of them, a myriad of graphical- and textual- based tools has been developed with the aim to simultaneously make the process of networks design increasingly more intuitive and to speed up their functional description. In Sec. 2, we itemize the most representative tools and give some concise explanations of them.

Once a network is defined by its constituents and interactions, it is common practice to simulate the network by means of deterministic/stochastic solvers. The deterministic solvers cope with interlocking sets of differential or difference equations and require the specification of some initial conditions. Its response (*y*) is fixed, given the values of its input variables (*x_i_*), and implies that *var*(*y*|*x_i_*) = 0. This property distinguishes deterministic models from real-life experiments [17]. Stochastic solvers use pseudorandom numbers (treated as if they were random numbers distributed uniformly and indpendently) to produce different *y *values, starting from the same initial parameters. Hence, the response *y *is a random variable with *var*(*y*|*x*_*i*_) = *g*(*x*_*i*_), usually estimated through replication and fed with different random numbers. A user-defined accuracy level determines how many simulation runs are needed [18]. A stochastic trajectory or trace (corresponding to the output of a simulation run) is a sequence of temporal observations of the copy-number of the species in the state space.

Traces are curves generally made by double precision (64-bit) floating-point numbers sampled over time. They sketch the trend of some system variables that are changing because of a predefined set of mathematical rules. Based on the continuous or discrete nature of such rules, the traces format changes accordingly. Continuous traces exhibit smooth trends of variables regularly observed at continuous time-scales. The solution of an ODE-based biological system gives the trend of the concentration of their variables within a desired time interval. Therefore, variables change continuously according to a mathematical function which solves the ODE's set. Discrete traces only differ because of their non-homogeneous temporal sampling. They are evaluated at uneven time instants that are calculated at run-time. Usually, biological discrete systems deal with chemicals populations (in place of chemicals concentrations) and, consequently, Markovian trajectories are made of streams of integer numbers. Sharing both kinds of simulation results is, to a great extent, hindered by the use of a variety of data formats by the existing simulation software packages. In fact, most of them output traces decorated by proprietary meta-information that contains private, not human-readable simulator settings. Few standard formats exist, but although some of them seem to be very well-suited and complete, their general-purpose nature makes them little versatile for our needs. That is why *Snazer *has been equipped with an ad-hoc and lightweight internal data format.

The rest of the paper is organized as follows: Sec. 2 describes all the features of our tool compared to those of similar existing tools. Particularly, in Sec. 2.2 the graph layout algorithms and the analysis features of *Snazer *are presented, whereas in Sec. 2.3 we describe the implemented statistics routines. In Sec. 2.4, we present the internal data format together with the compression policy employed to enhance the storing of data. In Sec. 3 we test *Snazer *on a real case-study with the aim of highlighting features and strength points. Then, we perform some benchmark tests on its compression capability and show the results. In Sec. 4 we conclude the paper and present future works.

## Implementation

*Snazer *is a software prototype whose aim is threefold: (i) manipulating biological networks, (ii) providing advanced statistical analysis routines for simulated traces and (iii) improving the traces sharing and storing processes. *Snazer *can also be considered as a viewer package of *Beta Workbench *(BWB) [19]. By parsing its output files, *Snazer *imports two relevant pieces of information inherently bound to the modeled systems: the *graphs of the simulated reactions *and the *simulated traces*. It encloses them in a compact XML data structure, designed to work as an interchange data source/sink. Through this, networks and simulation data look tightly coupled.

**Figure 1:**
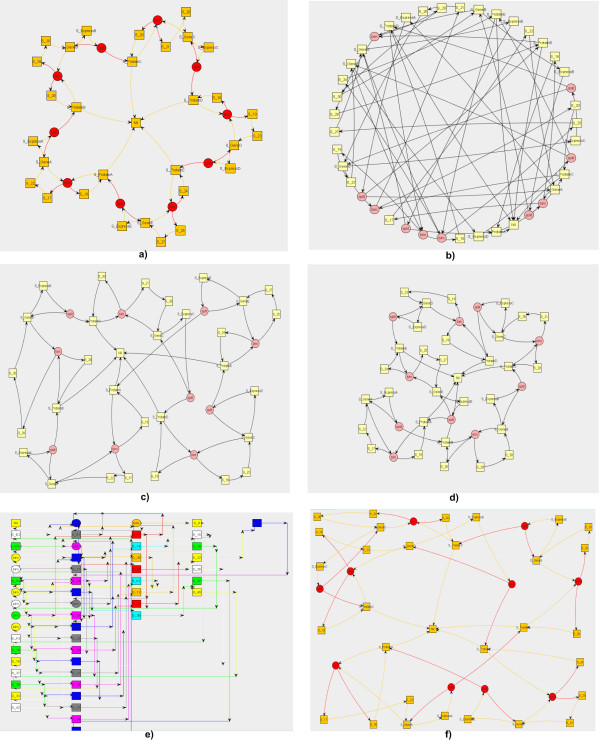
***Snazer *is made of two modules which dialogue by means of a shared, dedicated and XML-based structure**. We provide automatic conversion from/to some standard data formats.

The overall architectural skeleton draws inspiration from the Model-View-Controller software architectural design pattern and is made up of two parts: the *reaction graph *(Sec. 2.2) and the *statistics *(Sec. 2.3) modules, that deal with visualization of the system interactions and with calculation of statistics outcomes of simulated traces, respectively (see fig. 1). Both modules share the optimized floating object compliant with the *Snazer *XML-schema previously mentioned and discussed in Sec. 2.4 (cf. Additional Files 1).

### 2.1 State of the art

The rationale behind *Snazer *comes directly from the scientific community, which needs to manage together the biological models and their simulation results in a coherent manner. Several tools exist that unilaterally tackle such need. **Pajek **[20] is a standalone application for analysis and visualization of large networks having ten to hundreds of thousands of vertices. It is a highly interactive program and its main strength is the variety of layout routines (circular, energy, eigenvalues) that greatly facilitate exploration of patterns within large networks. It is equipped with several decomposition, connectivity, pattern searching algorithms. Pajek can detect clusters in a network, extract vertices that belong to the same clusters and show them separately, shrink vertices in clusters or show relationships among clusters. It loads several proprietary input files and writes network layouts as EPS, SVG, BMP files. **Medusa **[21] is a Java application (standalone or applet). It provides 2D representations of medium-sized networks, up to a few hundred nodes and edges. It shows multi-edge connections and is optimized for protein-protein interaction data. Medusa supports weighted graphs and represents the significance and importance of a connection by varying line thickness. Graphs can be drawn by means of some embedded spring-like layout algorithms and exported to image or postscript files. Medusa is designed for accessing protein interaction data from STRING[22]. **Cytoscape **[23] is a standalone Java application for the visualization of large-scale molecular interaction networks (directed, undirected and weighted) and their integration with gene expression profiles and other data. It also allows the manipulation and comparison of multiple networks. The great quantity of available plug-ins enriches Cytoscape with increasingly specialized analysis modules. The tool supports a variety of format files, including Gene Ontology (GO) [24], SBML[13] and KEGG[25]. **BioLayout Express**^3*D*^[26] is another tool written in Java for the visualization and analysis of networks derived from biological systems. It supports both unweighted and weighted graphs together with edge annotation of pairwise relationships. It mainly employs the Fruchterman-Rheingold [27] layout algorithm for 2D and 3D graph positioning and display and makes use of a heavily optimized C-based Markov Clustering algorithm for graph clustering. Its main goal is to offer different analytical approaches to microarray data analysis. It supports different file formats: Cytoscape Sif files, Reactome files [28], yEd GraphML files [29].

**Osprey **[30] visualizes complex interaction networks. It represents not only interactions in a flexible and rapidly expandable graphical format, but also provides options for functional comparisons between data sets. Osprey uses the General Repository for Interaction Datasets as a database (BioGRID) [31], from which the user can rapidly build interaction networks. Networks can be saved as tab-delimited text files for future manipulation or exported as JPEG, PNG, and SVG. **ProViz **[32] is a standalone open source application developed for the visualization of protein-protein interaction networks. It provides facilities for navigating large graphs and exploring biologically relevant features. It has a plug-in library with dozens of layout algorithms. Basically, they belong to three families: force-based, hierarchical and geometric layout (only circular). ProViz adopts emerging standards such as GO and PSI-MI[33]. **BiologicalNetworks **[34] models whole cell biochemical pathways and gene regulatory networks. It uses a specialized graph visualization engine to represent biological pathways, gene regulation networks and protein-protein interaction maps for intuitive exploration and prediction. The tool can handle a variety of tasks, including graphic drawing and layout optimization, data filtering and pathway expansion, and classification and prioritization of proteins. BiologicalNetworks uses a proprietary file format (BNX) that stores information pertaining to the model and the corresponding simulation environment. It supports import and export of models from SBML, SIF and GML file formats. Finally, **Tulip **[35] is one of the forerunners of drawing packages for biological networks. It allows the visualization, drawing and editing of graphs up to a million elements. Such a visualization system allows navigation through geometric operations as well as extraction of subgraphs and enhancement of the results obtained by filtering. Its most interesting property is the underlying data structure used to inspect huge graph attributes. Tulip implements the well-known "flyweight" and "chain of responsibility" patterns to access graphs through views. The real advantage is enabling a real sharing of the elements between graphs with a good memory management. All this software improve and obscure the first-generation tools from which they have drawn inspiration: **Otter **[36], a general-purpose network visualization tool; **Negopy **[37], a discrete, linkage-based program for the analysis of networks; **KrackPlot **[38], a network visualization tool intended for social networks; **MultiNet **[39], a Windows-based computer program designed for exploratory data analysis of social and other networks. Other tools exist that aim at providing advanced statistics routines for biological traces. **Traviando **[40] is a backend trace visualizer and analyzer. It interfaces the XML output file of M*ö*bius, a multi-paradigm multi-solution framework for the performance and dependability assessment of systems, to investigate the details of what happened in a simulation of a model. It verifies that certain events happen in a trace, that particular states are frequently reached or that certain conditions hold throughout a simulation. It further analyzes the cyclic behavior with graphics that show if states are repeatedly visited or how the length of the trace evolves if cycles are removed. Traviando also supports various statistics on traces as well as the model checking of a trace with respect to LTL formulas. **SimWiz **[41] is an old but still interesting project. It is a collection of Java tools that aims at visualizing data resulting from different kinds of biochemical simulation processes. It imports STODE[42] and COPASI[43] simulation output files as well as the relative reaction graphs as SBML files. Its main feature is animating the network graph through the information coming from the simulated traces. **VANTED **[44] loads and edits graphs, which may represent biological pathways or functional hierarchies. It allows the mapping of experimental data sets onto the graph elements and visualizes time series data or data of different genotypes or environmental conditions in the context of the underlying biological processes. Built-in statistic functions allow a fast evaluation of the data (e.g. t-Test or correlation analysis). **PopTools **[45] is a versatile add-in for Microsoft Excel that facilitates analysis of matrix population models and simulation of stochastic processes. Together with routines for iterating and resampling, this allows the calculation of bootstrap and other statistics for stochastic processes. Routines that facilitate calculation of some simple maximum likelihood and resampling statistics are supported as well.

*Snazer *addresses many of these issues, but additionally offers a solid framework that couples networks and traces analysis frameworks. It provides the user with the possibility both of browsing, dissecting and analyzing the networks components and of performing statistics on the inspected components. *Snazer *has been equipped with an efficient compression system to make huge amount of data manageable and treatable as well as to store and share them. Compared to the existing tools, it covers most of the network visualization layout routines. In addition, it implements Temporal-Circuit (see Sec. 2.2), an original algorithm to layout networks according to the information coming from simulation. It is not as well equipped with decomposition and connectivity algorithms as Pajek and BioLayout Express^3*D*^, because we mainly focused on original (e.g. ColorBlind filter and standard annotation inspection - see Sec. 2.2.2) rather than existing functionalities. *Snazer *is cross-platform, being implemented in Java (like Medusa, Cytoscape and BioLayout Express^3*D*^). It structures input data in XML files (as BiologicalNetworks does) and makes use of an internal data structure to improve data access (like Tulip). At the best of our knowledge, *Snazer *is the only tool that provides data compression (Sec. 2.4). It supports some standard input data formats (as Medusa, Cytoscape, BioLayout Express^3*D*^, ProViz and BiologicalNetworks) to be fully compatible with all the orbiting software packages. Moreover, *Snazer *offers some new trace analysis routines that deeply differ from those implemented in the presented softwares. In fact, VANTED fills network nodes with information coming from biological experiments (like microarray, proteomics, etc) to statistically analyze them. But it does not provide any means to analyze simulated traces. SimWiz, instead, makes use of traces to animate the related networks, but it does not provide any functionality in support of simulation. Traviando analyzes individual traces by using classical model-checking methods, but lacks of any support for group-traces. PopTools, offers some statistical facilities for wet-data (as VANTED) and stochastic processes, but disregards any group-traces issue as well. Contrarily, *Snazer *covers these lacks by offering new analysis algorithms centered on multi-traces analysis.

All these features are built-in in *Snazer *and make it a unique tool.

### 2.2 The reaction graph perspective

The focus of network-level analysis in general is on properties of networks as a whole. These may reflect, e.g. typical or atypical traits relative to an application domain or similarities occurring in networks of entirely different origin. Both from a global and a local view, quantitative analyses have been conducted on networks to investigate these traits. *Snazer *provides the user with some clever layout algorithms to firstly give a global insight of a network, and with a *centrality *ranker index to highlight vertex importance.

#### 2.2.1 Layout analysis

Graph layout methods are core techniques for applications based on graph-like network diagrams. Since such a perspective is becoming more and more pervasive, and since *Snazer *visualizes part of the data sets as reaction graphs, we equipped it with the fastest and most flexible layout algorithms. Most of them belong to the *force-based *(or *force-directed*) class of algorithms. They are easy to use, since they do not require special knowledge about the graph theory, and often their results look very good. They mainly assign forces to networks as if the nodes were electrically charged particles and the edges were springs. Thus, layouts are arranged according to real physical principles (e.g. Coulomb's law, Hooke's law, etc). As a result, forces repetitively applied to nodes push and pull them further apart in order to minimize the overall energy of the networks. When an equilibrium state is reached, the graph is drawn.

**Figure 2:**
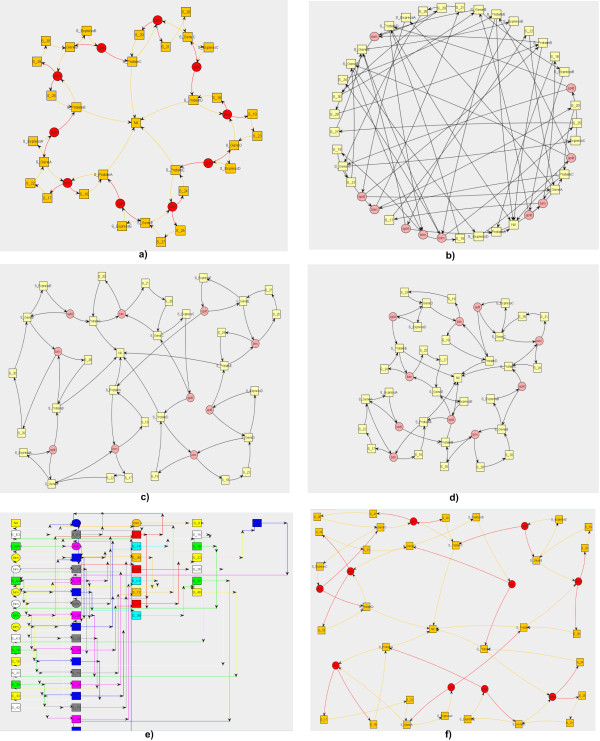
**a) Kamada-Kawai, b) Circle, c) ISOM, d) Spring, e) Temporal Circuit, f) Fruchterman-Reingold**.

Such algorithms enjoy several strength-points: *uniform nodes distribution*, *symmetry*, *flexibility*, *simplicity*, *strong theoretical foundations*, but suffer from some drawbacks: *high running time *(equivalent to *O*(*V *^3^)) and *poor local minima*. *Spring *is the simplest *force-based *layout algorithm. Basically, nodes try to get as far of each other as possible, but edges pull nodes near each other, merely according to their *weights*. The energy of the system is minimized by iteratively arranging the nodes (fig. 2d). It enjoys all the strength-points as well as it suffers from all the drawbacks just mentioned.

In particular, the task of bringing under control the problem of poor local minima raised the interest of many scientists. Such issue is based on the fact that the obtained minimum system energy could be considerably worse than a global minimum. Since such issue becomes more and more important as the number of vertices of the graph increases and, hence, that the overall quality of the drawing could result lower and lower, some scientists focused on the *initial layout *sub-problem to solve it. They conjectured that any outcome of any force-based algorithms results to be strongly influenced by the initial layout, that in most cases is randomly generated.

The *Kamada-Kawai *(KKLayout) algorithm represents the first attempt to solve this problem. It was designed with the aim to quickly generate advantageous initial configurations. Nodes are represented by steel rings and the edges are springs between them. The attractive force is analogous to the spring force and the repulsive one is analogous to the electrical force. The energy minimization in this algorithm is achieved by obtaining the derivative of the force equations. At the minimum energy, the derivatives of the force equations are zero. Since these equations are not independent, they cannot be independently brought to zero, and therefore, only the node that has the maximum gradient value is moved. This process is repeated until the total energy is minimized (fig. 2a).

A combined application of KKLayout with the *Fruchterman-Reingold *(FRLayout) algorithm represents today one of the most successful layout solutions. FRLayout contributes in meaningfully placing the neighbored nodes. It works with unweighted, undirected graphs, where attractive forces occur between adjacent nodes only, and repulsive forces occur between every pair of nodes. The movement of nodes is also function of the system temperature registered at each iteration. Generally, temperature decreases through successive iterations while nodes occupy their place (fig. 2f).

Some alternative ways of approaching this problem lie in searching more directly for the energy minimum, either instead of or in conjunction with physical simulation. Among such procedures, we consider a competitive learning method: the *ISOM *layout (ISOMLayout), which extends the *Kohonen*'s self-organizing map. It evenly fills the space with vertices and lets them take place because of an heuristic function rather than of attracting forces. The algorithm selects a random point in the graph area and picks the closest vertex to that point. This vertex is moved toward that point as well as all vertices connected to that initial vertex by up to a set number of edge steps. The amount by which the vertices are moved decreases the greater the number of edges in the shortest path between the current and initial vertices. The initial number of edge steps is decreased during the layout process so that the later steps form local clusters of connected vertices (fig. 2c).

Unfortunately, such methodologies do not properly work with huge networks, where problems like node-node or node-edge clashes are often encountered. In these cases, simple algorithms like the *Circle *layout are employed with the aim to evenly space vertices on a geometric (circular, in this case) trajectory, irrespective of the network size. *Circle *attempts to minimize as many overlapping vertices as it can, by placing vertices next to each other that are adjacent in the graph. It is a fast algorithm, essentially because it is not optimal. That is, it does not resolve the problem of edge intersection, but propose a more familiar way to visualize the network. It does not undergo sequential arrangement since it is a static layout (fig. 2b). All the described layouts are general purpose and have been widely employed in heterogeneous scientific areas. We designed and implemented *Temporal-Circuit*, an original and ad-hoc layout algorithm, aimed at drawing simulated biological networks. It locates network elements in an electric circuit. Nodes and reactions take progressively place into tabular spots from left to right. In particular, the species initially present into the system and the reactions which involve them are drawn on the leftmost. Afterward, if new species are created somewhere and somehow into the system (e.g. because of some synthesis reactions), they are depicted on the immediate right, together with the reactions which implicate them. This process progressively continues until any new species is placed. Therefore, the species that come last into the system occupy the rightmost side (fig. 2e). Since such a layout encodes and visualizes both the network and simulation information, it works only if both the graph of reactions and the simulated traces are provided by the user.

#### 2.2.2 Quantitative analyses

The determination of important elements, group of elements or evident treats of networks is collectively known as quantitative analysis [46]. Since the 1950s, along with some global topological indices, many vertex centrality indices were introduced. These were to quantify an intuitive feeling that in most networks some vertices and edges are more central than other. In practice, they were to evaluate the 'reachability' of a vertex.

Given any network, these measures rank the vertices according to the number of neighbors or to the cost it takes to reach all the other vertices from it. These centralities are directly based on the notion of distances within a graph, or on the notion of neighborhood, as in the case of the degree centrality. *Degree*, *eccentricity*, *closeness*, *centroid *are only some among the most known and simple existing centrality indices. On their basis, several structural properties have been studied, e.g. the graph *center*, defined as the set of all the vertices of minimum eccentricity; the *median *graph, an undirected graph in which any three vertices *a*, *b*, and *c *have a unique median: a vertex *m*(*a*, *b*, *c*) that belongs to shortest paths between any two of *a*, *b*, and *c*.

Based on the set of shortest path in a graph, some other centrality indices are worth being mentioned: *stress centrality*, that is based on the enumeration of shortest paths; *shortest-path betweenness centrality *is a kind of stress centrality that accounts for the fraction of shortest paths between two nodes that contain a third node.

Vitality measures have been further used to determine the importance of vertices or edges in a graph. Given an arbitrary real-valued function on a graph, a vitality measure quantifies the difference between the value on the graph with or without the vertex or the edge. In particular, *flow betweenness vitality *is a measure for max-flow networks which is similar to the shortest-path betweenness, but that aims at measuring the degree that the maximum flow depends on a particular vertex; *closeness vitality *denotes instead how much the transport cost in an all-to-all communication will increase if a vertex is removed from the graph.

These and all the other existing local methods try to answer to questions like: *which are the most central members of a network and which the most peripheral? Which connections are crucial for the functioning of a subnetwork?*

*Snazer *contributes to answer to these questions by providing a little set of interactive tools. Mainly, a *degree *ranker is implemented with the aim to highlight centrality of nodes within graphs. Ranks are calculated according to the corresponding nodes degree and, then, nodes color tonalities are tuned accordingly: the more a node is central, the darker will be its color tonality. Degree centrality is meant as the number of links incident upon a node (i.e., the number of ties that a node has). Degree is often interpreted in terms of the immediate risk of node for catching whatever is flowing through the network (such as a virus, or some information). If the network is directed (meaning that ties have direction), then we usually define two separate measures of degree centrality, namely *indegree *and *outdegree*. Indegree is a count of the number of ties directed to the node, and outdegree is the number of ties that the node directs to others. For positive relations such as friendship or advice, we normally interpret indegree as a form of popularity, and outdegree as gregariousness.

Mathematically, for a graph *G *with *n *vertices, the degree centrality *C_D_*(*v*) for a vertex *v *is defined as:(1)

**Figure 3:**
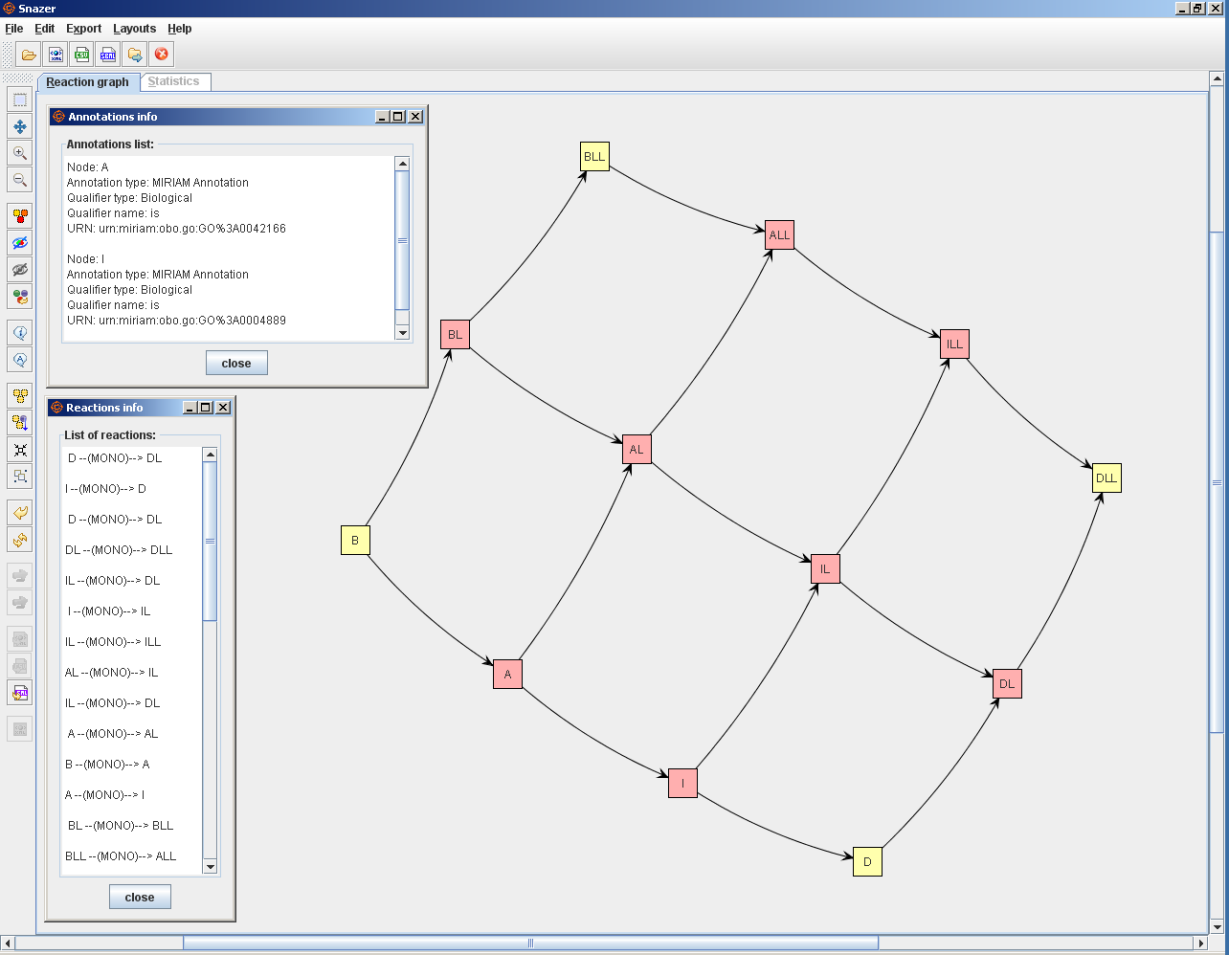
**An example of **MIRIAM** annotations (top-left) and of reaction information (bottom-left)**.

Each graph drawn by *Snazer *is fully interactive. They can be graphically modified by stretching, translating and grouping nodes and can be saved as standard GraphML files. *Snazer *provides users with the possibility to discover any information embedded into nodes and edges. If encoded in a standard manner, nodes hold data source information in the form of MIRIAM annotations [47]. By them, any detail (official name and synonyms, root URI, pattern of identifiers, documentation, etc.) refers to a catalog of data types through URIs and to their physical locations through URLs. On the contrary, edges carry information about the nature of the reactions that they represent (e.g. monomolecular, bimolecular, degradation, etc.). An example is reported in fig. 3.

Another important feature is the support for color-blind users. *Snazer *takes care of the most common color vision deficiencies (protanopy and deuteranopy) that affect the 8% of the male and the 2% of the female populations [48]. The set of the graph colors can be tuned on-demand by means of our own procedure, referred to as *ColorBlind filter *[48]. It provides users with a sufficient color contrast between nodes and the foreground/background, by changing the *hue*, *saturation *and *lightness *values of each color in a proportional way.

In any case, nodes can be visited and highlighted by the intuitive relationships of *neighborhood*, or by user selection. Selected nodes can be sent to the analysis module to be further analyzed.

### 2.3 Statistical perspective

Many features have been ascribed to simulation traces over the years: (i) the *trend component*, namely the long term underlying direction (an upward or downward tendency) and rate of change; (ii) the *irregular component *(or 'noise'), namely the component that is left over when the other components of the series have been accounted for; (iii) the *autocorrelation*, namely the relationship between members of a time series of observations and the same values at a fixed time interval later. Some scientists make use of specialized algorithms to look for these and others properties in sets of traces, obtained by repetitive simulation of the same models. In their most basic form, multiple traces analysis algorithms treat all variables symmetrically without making reference to the issue of dependence versus independence and permit causality testing of all variables simultaneously. This is a major advantage of such algorithms compared to the multivariate time series algorithms. On the basis of these algorithms, we equipped *Snazer *with 7 analyzers, dealing with isolated as well as grouped time series, each with its own specific parameters.

They all tackle the common problem of dealing with differently sampled traces. This problem is essentially due to the mathematics behind the generation of the time vector. Indeed, whenever a Gillespie-inspired algorithm for simulating a model is used, the time evolution of a well-stirred set {*S*_1_, ..., *S*_*N*_} of biochemical species reacting through *M *≥ 1 reaction channels (*reactions *the hereafter) {*R*_1_, ..., *R*_*M*_} depends on:

• the probability *a*_*j*_()*dt *that, given , one reaction *R*_*j *_will occur in the next infinitesimal interval [*t, t *+ *dt*). N.b. *a*_*j*_() is a function of the number of possible active instances of reaction *R*_*j*_. Consider a reaction *R *: *S*_1 _→ *S*_2_, then *a*() = *cx*_1_, where *x*_1 _is the number of active *R *in the current state , and *c *is a constant that depends on the physical characteristics of *S*_1_;

• the change *v*_*ji *_of the number of molecules of the specie *S*_*i *_produced or consumed by a reaction *R*_*j*_.

**Figure 4:**
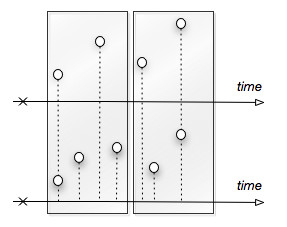
**Often stochastic traces obtained by MRiP calculations exhibit different sizes and warped time vectors**. Alignment procedures, like *binning*, are widely used to re-sample traces and align the corresponding values.

Given *a*_*j*_(), the evolution of a biochemical network is described by the *next reaction density function p*(*τ*, *j*|, *t*), which represents the probability, given , that the next reaction in the system will occur in the infinitesimal time interval [*t *+ *τ, t *+ *τ *+ *dt*) and will be on channel *R*_*j*_.(2)

where . By conditional probability, Eq. 2 can be rewritten as  where(3)(4)

where *τ *is a sample from an exponential random variable with rate *a*_0_(), and the selected reaction *j *is independently taken from a discrete random variable with values in [1,M] and probabilities . By the Eq. 4 and Eq. 3, the next reaction and the instant of time when it will fire are then chosen. In particular, the repetitive calculation of the Eq. 3 provides the time vector of a simulation, whose sampling is strongly dependent upon the initial seed of the generated stream of *τ *values. Thus, in the context of a MRiP simulation [49,50], different initial seeds are carefully chosen and used to avoid the cross-correlation that would naturally exist between any pair of traces. However, although this does not represent a problem from a graphical point of view, traces with different sizes and different time scales would result difficultly comparable from an analytical perspective. E.g., in the case of two traces with different samplings (as in fig. 4), a slightly warping effect might be noticed at a certain point in their time scales. This means that it might happen that some corresponding points might be plotted on shifted temporal locations and, hence, that even the simplest analysis task might fail on them.

Our analysis routines overcome this issue. They all are equipped with an embedded *pre-processing *functionality that prepares traces for analysis. It takes a set of traces and applies an alignment procedure called *binning *[51-54], which has a twofold effect. It re-samples traces and meaningfully reduces their sizes. Binning is one of the most used pre-processing technique in the area of signal analysis. It groups adjacent points into *bins *and elects a representative member for each group. In particular, it takes a subset of *N *points from a generic signal, represented by the couples [(*v*_1,_*t*_1_), (*v*_2,_*t*_2_) ..., (*v*_*N*,_*t*_*N*_)], and substitutes them with an unique point (v*, t), whose value v* is an aggregate function of the *N *original values (e.g. their sum), and the time *t *is usually chosen among the original times (e.g. as the median time, or the time corresponding to the maximum value). Such basic operations are conducted by scanning all the signal through a sort of sliding window (of fixed width) chosen by the user (Here, it should be noted from fig. 4, a constant window could contain a variable number of points along a signal).

This technique has been successfully used in the area of *Mass Spectrometry *time course analysis, where the time warping problem equally exists.

#### 2.3.1 Analysis routines

• *Series *allows the application of a plethora of statistical calculations to traces. It elaborates an output data point for each *timestamp *present in any of the input traces and produces a *time series *as output, one for each selected statistical routine and for each selected chemical. The available statistical calculations are: *mean, root mean square, variance, standard deviation, standard error, geometric mean, harmonic mean, skew and kurtosis*.

• The *first hitting *analyzer works like *series*. However, it samples traces only whenever a (user-defined) boolean condition becomes true. Indeed, for each run, it searches for the first timestamp where the condition is satisfied and performs the requested statistics on the selected species. Finally, it outputs the result as points on 2D charts, where time runs on the *X *axis.

• *Pointwise *is a uniform analyzer which differs from *series *only because it performs whatever chosen statistics at regular, user-defined, intervals.

• The *steady state *analyzer is applied to systems characterized by initial transient behaviors. Under the assumption that the chance of entering whatever possible state of the system after a transient period is time-independent, this analyzer chooses a random timestamp (after the transient time) from each run, and performs there the requested statistics.

• The *cumulative *analyzer calculates the maximum, minimum and mean (as the integral of a trace divided by the total time) values for each selected species within all the time intervals that verify a pre-defined boolean condition. In other words, it gives the possibility of performing statistics only within some limited slices of traces.

• The *time *analyzer measures the duration of the time intervals that verify a user-defined condition. We call "front" the timestamp when a condition becomes true (or vice-versa). Fronts can ascend or descend, whenever the related conditions flip from false to true (and vice-versa). Thus, this analyzer performs statistics between any two consecutive fronts. Other than the usual statistics, it can further perform *time-duration *and *time-sum *calculations.

• The *raw data *analyzer displays data on the 2D chart, as they are.

*Snazer *gives the user the possibility to alternatively display results in the chart panel at run-time or to export them in SVG file format.

### 2.4 Data boxing

As quickly introduced above, *Snazer *makes use of an internal data representation. Before choosing it as our favorite data structure, we examined the three major alternatives:

• HDF (Hierarchical Data Format [55]) is designed to assist users in the storage and manipulation of scientific data across diverse operating systems and machines. It is generally employed for managing very large (and complex) data sets with very fast access requirements. Its main aim is to standardize the format and descriptions of many types of commonly used data sets (such as computerized images and scientific data). It allows self description of data and accommodation for symbolic, numerical and graphical information. It is platform independent.

• NetCDF (Network Common Data Form [56]) is a data format made for managing array-oriented scientific data. As HDF, it is a standard, platform-independent format which allows self description of data and efficient information retrieving. It makes use of HDF to enhance managing of much larger files and multiple unlimited dimensions.

• SBRML (Systems Biology Result Markup Language [57]) is a proposal for a new markup language whose aim is to give structure to the results of typical operations performed on SBML models. SBRML captures and describes operations, type of operations and the algorithms used to perform such operations. It is currently structured on three levels: (i) ontology terms, (ii) software, algorithm and result information and (iii) content of the result.

Unfortunately, all three data formats did not properly suite our aims. The first two are very general-purpose and too complex for us. In fact, we only need to efficiently store/retrieve time course data and simultaneously keep them linked with the corresponding biological models. SBRML could work, but because it is still a proposal and it lacks of guidelines, it has never been adopted on concrete case-studies. Consequently, we discarded it as well.

#### 2.4.1 The internal data format

The internal data format is a trade-off between simplicity and functionality. It is ad-hoc made and it has not been meant to compete with the existing data formats. It makes the dialogue between both the visualization and the analysis modules possible. It is an intermediate and XML-encoded data structure (cf. Additional File 1). To be coherent with the content of both modules, it is logically structured in two tightly coupled sub-parts. The former takes inspiration from SBML in both inheriting and specializing some of its constructs. Thus, since *Snazer *agrees on the same policies of annotation and identification, the SBase and SId elements are inherited as-they-are. Furthermore, whichever biological system is specified by a model entity and by a list of reactions, each containing a list of reactants, of products and of their chemical kinetics rates. Differently from SBML, each reaction is categorized by a *type *tag. Reactants and products reference three types of chemicals: entities, complexes and variables. The first two types allow representation of structured elementary or complex reactive chemicals. Variables are destructured elements which express scalar measurements (*mass, temperature, density, volume, etc*.) evolving over time.

The remaining part of the schema captures the information concerning the simulated traces and time. In particular, a (de)structured chemicals is here referenced and annotated by means of the *tstart *attribute, which specifies the time when it enters into the system, and of the *structure *attribute, which records its internal behavior (if any). Finally, streams of simulated timesteps are compressed and recorded together with the information about the first, last simulated timesteps and the overall number of timesteps. Traces are equally sampled over time.

#### 2.4.2 The compression policy

**Figure 5:**
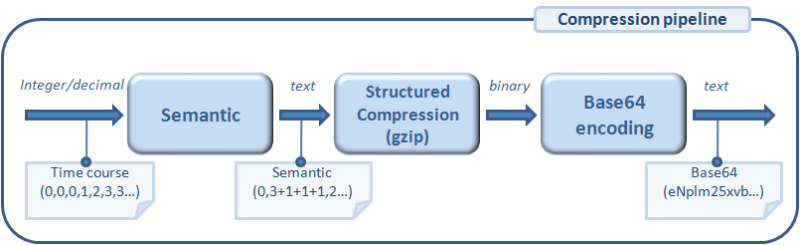
**(From left to right) A flow of integer or decimal numbers is given in input**. A stream of textual tuples is generated by a semantic compressor. Then, it flows in input to a structural compressor based on the *gzip *algorithm which further compresses and transforms the textual stream into a binary one. It is finally encoded into a printable Base64 stream of characters to be recorded into a XML file.

Compression is one of *Snazer*'s strength points. Traces of both integer (entities or complexes population changes) and decimal (variables changes) numbers streams are compressed by applying a pipeline of *semantic *and *structural *compressors. The simple semantic compressor takes in input a stream of numbers and outputs a stream of tuples (*value*, *persistence *'+' increment*), where *value *is the placeholder for any number of the input stream, *persistence *counts the simulated instants of time where *value *stays constant and *increment *stands for a list of (relative) changes from *value*, for each number of the input stream subsequent to *value*. A new tuple is produced whenever one of the numbers of the input stream stays constant. The overall stream of tuples flows into a structural compressor where it is further compressed by the *gzip *algorithm. As a result, an ultra-optimized binary stream is produced. With the final aim to write it to file, it is transformed into a printable (MIME) *Base64 *string (see fig. 5).

## Results

We successfully used *Snazer *to analyze many real case-studies (some of them are enclosed in the Additional File 2). Here we make use of a couple of them to show some of its functionalities. They are the *cell cycle *[58] and the *circadian clock *[59] mammalian models. The former is a complex network of biochemical phenomena that controls the duplication of cells. Such process is macroscopically subdivided into four phases which cyclically alternate because of some cyclin-dependent protein kinases (CDKs). When bound to specific cyclin partners, CDKs promote cellular progression along the cellular life-cycle. The latter model reproduces periodic critical triggering signals in charge to control the cellular size during the cell cycle. Merged together, they give raise to a very interesting model [60].

**Figure 6:**
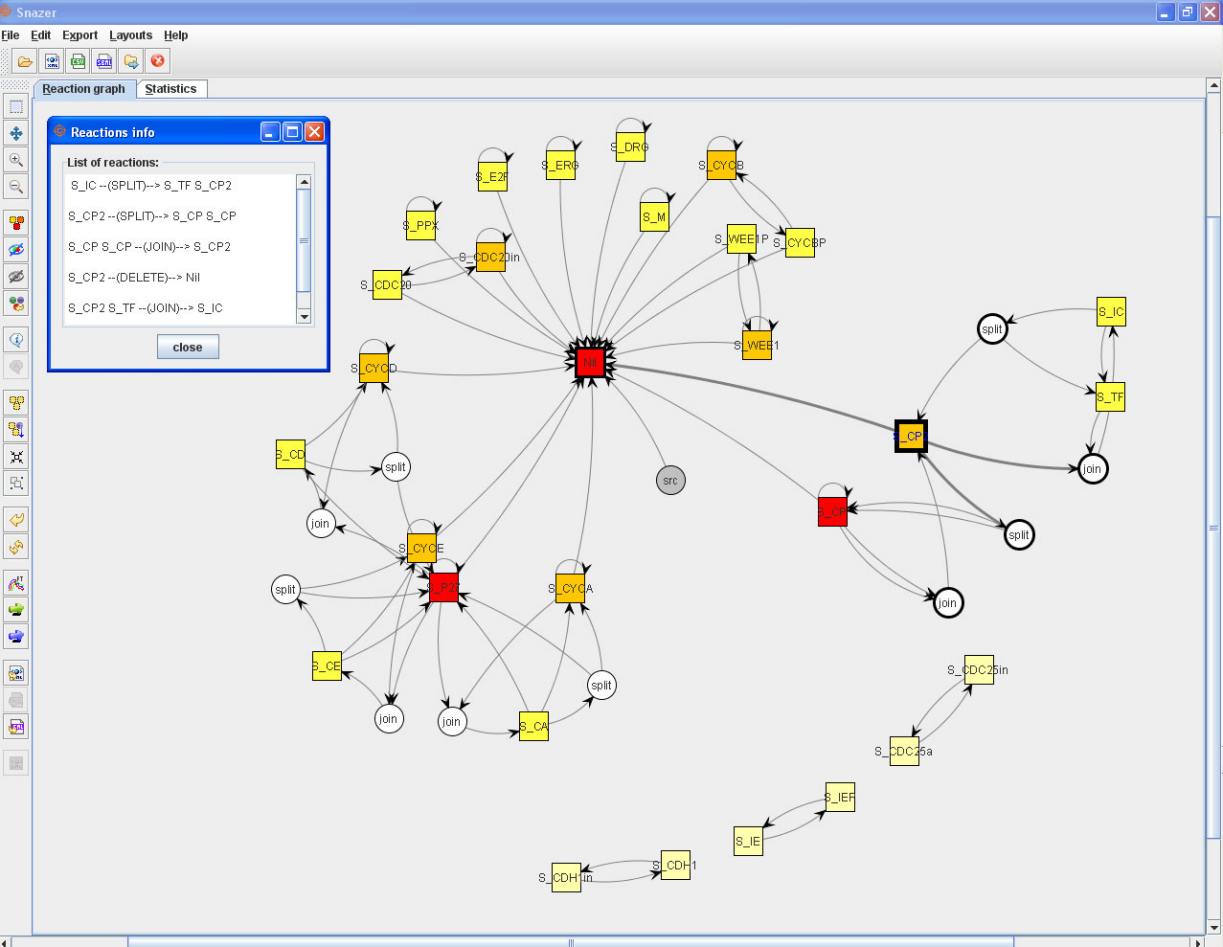
**The graph is composed by two sub-graphs**. On the left is the cell cycle model, on the right the circadian clock one. Both are connected by 2 (logical) links. By applying a node importance ranker routine, S_CP is found to be one of the most *central *nodes within the graph. We list all the reactions in which it is involved (top-left).

By applying the *Kamada-Kawai *layout algorithm (cf. Sec. 2.2 and see fig. 6), the modular feature of this network looks very evident. A fair chunk of it shows the cell cycle part (on the left side). The other accounts for the circadian clock part. Both are not explicitly linked, but they are dependent because of the influencing activity of the S_TF clock component on S_Wee1. The used layout algorithm manages to catch this aspect. Once drawn the network, we estimate the *importance *of the nodes by running a *degree ranker*. As a result, it changes the nodes color tonalities as follows: the lower the nodes hue, the lower their importance within the network. Finally, we list all the reactions which S_CP2 (the rightmost orange component in the figure) is involved in. By just a click on it, its neighbor nodes and their connecting edges get highlighted (i.e. their borders become thicker) and selected. The selected nodes can be further analyzed or passed to the statistics panel.

The statistics panel turns on whenever at least one trace (with chemicals labels matching the network nodes labels) is imported. We used three different software packages to perform multiple simulations of the model: BWB [19], Cyto-Sim [61] and COPASI [43].

**Figure 7:**
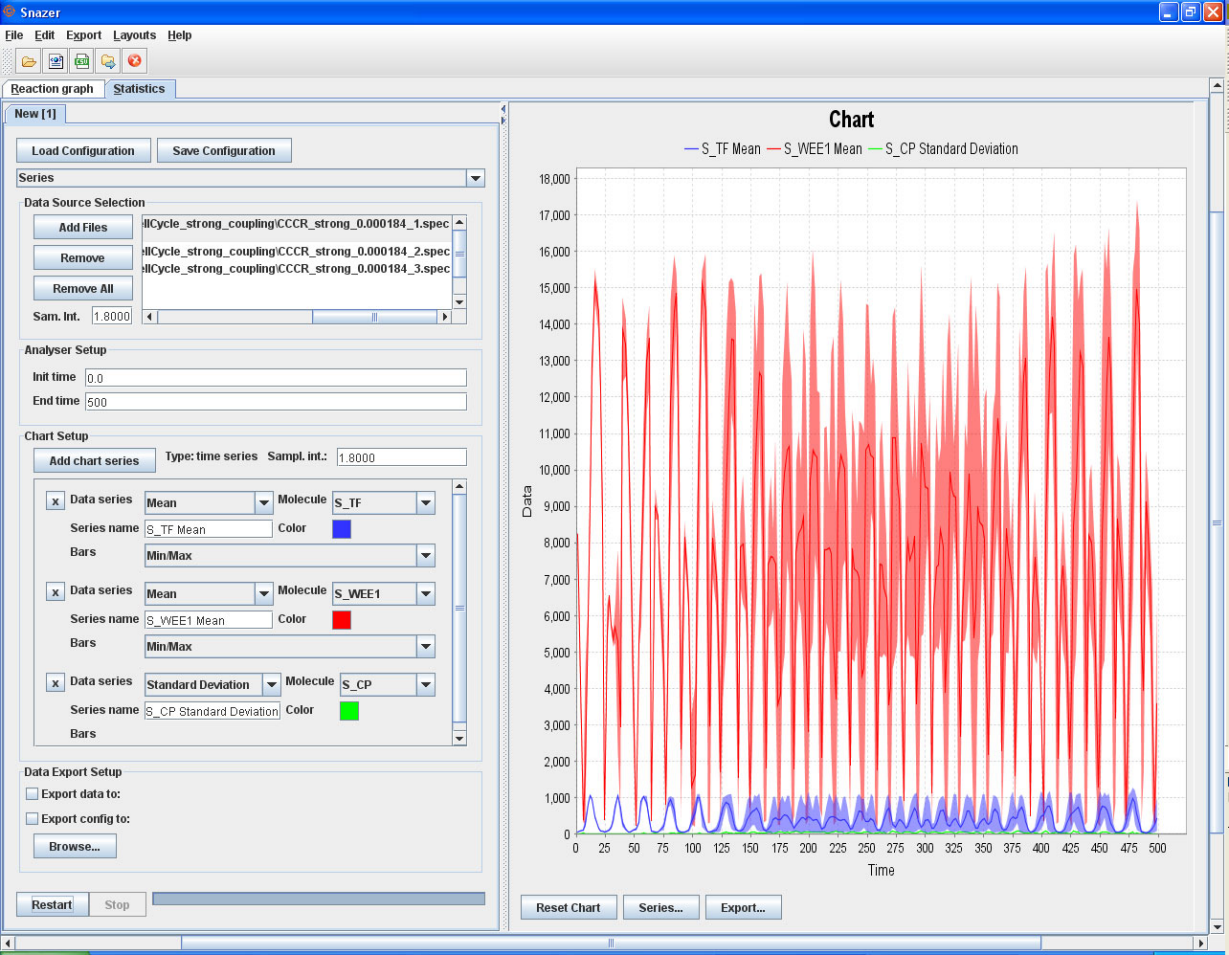
**The time analyzer applied on the case-study**.

Initially, we import their traces and adopt the *time *analyzer. This is usually used whenever the user wants to count the number of oscillation periods (of the concentration or population size) of a species, or to estimate the regularity of the cycles, or to measure the duration of the time intervals when a species is over/under a threshold. One can specify an (even complex) condition to be satisfied by one or more species. For each species, one can specify the *front *type (cf. Sec. 2.3) and the x-axis point where to print the results. Since by means of the *raw data *analyzer we have verified in advance that S_TF oscillates around the value 600, we are able to quantify the duration of its period by measuring the time intervals between two consecutive values 600 (e.g. ascending fronts). In fig. 7a, the number of detected periods and their durations are shown in red. Instead, the duration of any interval between two consecutive ascending and descending fronts is shown in blue and green. In this case, we measure the time interval during which S_WEE1 and S_CP are below the specified threshold.

**Figure 8:**
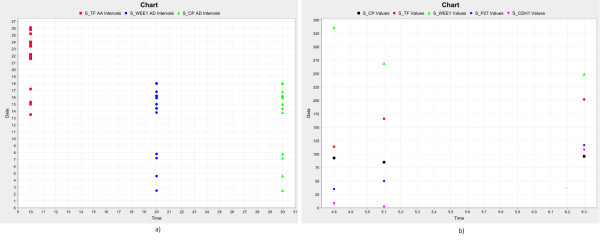
**We have applied the series analyzer to three multi-simulations of the cell cycle - circadian clock coupled model**. We set the plotting range between 0 and 500 and have picked up S_TF, S_WEE1 and S_CP as the species of which performing the *mean *and the *standard deviation *statistics. We have requested the plotting of the *variance bars *as well.

Another example lies in calculating a particular statistics (e.g. *mean *and *standard deviation*) exactly and only when a condition becomes verified (we make use of the *variance bars *to highlight the stochastic difference among all the simulated traces, see fig. 8). To this aim, we use the *first hitting *analyzer and we define a *two-values condition *that imposes that a molecule quantity (S_CP) is less than another (S_TF). Hence, by applying the analyzer, one of the mentioned statistics is computed on the selected species whenever the condition becomes verified (fig. 7b).

### 3.1 Benchmark tests

We performed some benchmark tests about the *Snazer*'s ability to compress input data. We also tested the memory allocation due to the XML-encapsulation process. Results are shown in Table 1. We simulated some biological models and saved their traces as CSV files. Subsequently, we inflated them using our compression pipeline and saved them in XML format.

**Table 1:** 

Xml-incapsulation routine benchmarks			
**Name**	**BWB**	**XML (compression %)**	**Allocated memory**

CC(1)	55.619	7.974 (85.66%)	398.248
UAT(1)	68.276	20.486 (70.00%)	403.136
CC(2)	102.870	19.875 (80.68%)	503.344
UAT(2)	106.271	21.100 (80.15%)	480.448
MAPKc	577.962	50.618 (91.24%)	1.721.616
Bycc(1)	909.064	172.266 (81.05%)	2.038.944
Gpc-RS	8.293.036	190.131 (97.71%)	21.790.768
Bycc(2)	130.821.452	3.735.872 (97.14%)	241.635.920
Bycc(3)	136.219.582	3.913.680 (97.13%)	191.823.072
Bycc(4)	148.322.373	4.318.744 (97.09%)	192.881.840

Data set names have been shortened in: *CC *(Circadian Clock) [59], *UAT *(Unpacked antagonistic toggle switch in budding yeast cell cycle) [62], *MAPKc *(MAPK cascade), *Gpc-RS *(G-protein-coupled receptor signaling) [63] and *Bycc *(Budding yeast cell cycle) [64]. Measurements are expressed in KByte. We list the names of the models in the first column. The second one identifies the traces files size generated by BWB. The third one holds the sizes of the corresponding compressed XML files (in percentage). The last one lists the quantity of memory allocated (in KBytes) for the compression and conversion routines from BWB to XML formats.

The compression routine results to be more effective the larger the model and the data set. In particular, it works fine on stochastic and integer traces, essentially because any sequential and stochastic framework schedules always only one reaction to occur at a time, while all the others stay unchanged. Thus, since reactions occur because of their own propensity functions, some will fire more frequently than others and, hence, models with many reactions will generate some frequently changing traces and some rarely changing traces. Moreover, the probability for a reaction to stay unchanged generally increases with the number of the reactions, irrespective of its propensity function.

The semantic compressor defined in Sec. 2.4 works fine when reactions do not change frequently, since all the consecutive stationary values are embodied by just one *persistence *value. Moreover, the best case occurs when traces are made of integer numbers. In that case, the *increment *part of the tuple will be always made up of a few figures (stochastic frameworks based on SSA deal with molecular reactions which involve few species) and the resulting compressed string will be the shortest. Contrarily, traces of decimal numbers would see the *increment *value always made of as many figures as the numbers precision.

**Figure 9:**
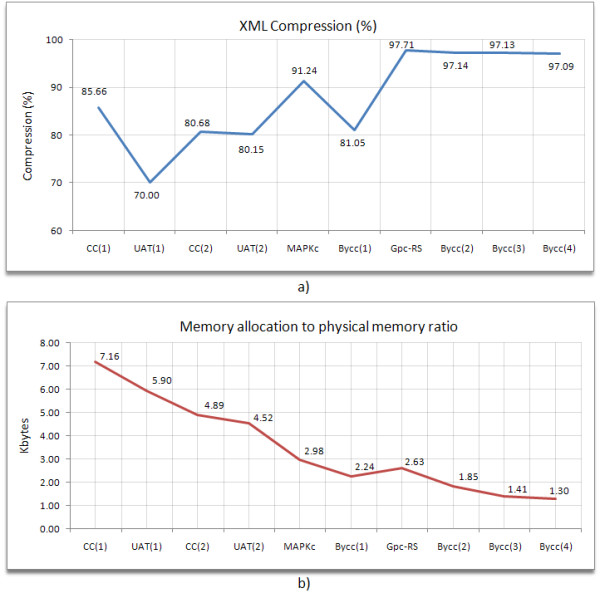
a) Benchmark tests of *Snazer*'s ability to compress simulated traces (see also Table 1), b) Ratio between the memory allocated because of the fixed overhead of the internal XML format and the effective size of each model.

Therefore, the resulting compressed string will be longer than one of the same length, but made of integers. Generally, we detected a slight increase in terms of memory usage due to the fixed overhead introduced by the XML structure. However, the larger the data set, the more negligible its overhead. This is shown in fig. 9b, where we depicted the ratio between the (fixed) memory allocated to perform the boxing process and the physical input file sizes. The curve goes significantly down with the increasing of the input file sizes.

## Conclusions

We implemented *Snazer *to help visualize biological networks and to handle and store their simulation traces. In particular, *Snazer *has been equipped with two main categories of tools. The visualization tools are in charge of drawing graphs according to some well-known as well as new layout algorithms. The new *Circuit layout *has been presented in this context. Furthermore, some quantitative analyses routines have been implemented to allow the user to investigate some common traits of the biological graphs as (e.g.) *node centrality *and *node importance*. On the other hand, *Snazer *has been supplied with a set of statistics routines with the aim to analyze the time course files obtained by simulation. Finally, *Snazer *relies on a very efficient compression routine that allows for comfortable storing and sharing of the simulation results. We plan to extend *Snazer *with further capabilities, such as remote database interfacing for data collection, more sophisticated graph *centrality *calculations and analysis routines (e.g. Fast Fourier Transform). We finally aim at increasing compatibility with other standard languages (e.g. CellML) and I/O data formats.

## Availability and requirements

Project name: *Snazer*; Project home page: https://www.cosbi.eu/index.php/research/prototypes/snazer; Operating system(s): Platform independent; Programming language: Java; Other requirements: Java 1.6 or higher, libSBML 3.3.1 or higher; Upon acceptance of the CoSBi-SSLA license, *Snazer *is freely available for non commercial purposes.

## Authors' contributions

TM conceived of the study, supervised its implementation and drafted the manuscript. GI implemented the most parts of *Snazer*. CP participated in the coordination of the study. All authors read and approved the final manuscript.

## Supplementary Material

Additional file 1 **Snazer XML schema**. The XSD file and two explicative JPG and HTML files. (*snazer.zip*) Click here for file

Additional file 2 **Example models**. Some example models. (*examples.zip*) Click here for file
